# Genetic variations in *TERC* and *TERT* genes are associated with renal cell carcinoma risk in a Chinese Han population

**DOI:** 10.18632/oncotarget.20163

**Published:** 2017-08-10

**Authors:** Dapeng Wu, Guodong Zhu, Jin Zeng, Wenbin Song, Ke Wang, Xinyang Wang, Peng Guo, Dalin He

**Affiliations:** ^1^ Department of Urology, The First Affiliated Hospital of Xi’an Jiaotong University, Xi’an, Shaanxi, China; ^2^ Oncology Research Lab, Key Laboratory of Environment and Genes Related to Diseases, Ministry of Education, Xi’an, Shaanxi, China

**Keywords:** association study, single nucleotide polymorphism (SNP), renal cell carcinoma (RCC), *TERC*, *TERT*

## Abstract

Renal cell carcinoma (RCC) is a common malignant tumor of the urinary system, the pathogenesis of RCC is still unclear. It is reported that genetic variations in telomere length related-genes *TERT* and *TERC* are involved in the many types of cancers. However, little is known about the association between *TERT* and *TERC* polymorphisms and susceptibility to RCC risk. To solve this problem, a total of 293 patients with primary renal cell carcinoma and 459 healthy people were recruited in our study. Six SNPs of *TERC* and *TERT* were genotyped, and association analysis was performed. We found *TERC*-rs35073794 and *TERT*-rs10069690 were associated with an increased risk of RCC in an allele model. (OR =2.39, 95% CI = 0.99-5.80, p = 0.047; OR =1.39, 95% CI = 1.07-1.81, p = 0.014, respectively). The genotype “TC” of rs10069690 was associated with an increased risk of RCC in the genotype model. (OR =1.52, 95% CI = 1.11-2.08, p = 0.009).*TERC*-rs35073794 was associated with an increased risk of RCC in the codominant model. (OR =2.61, 95% CI = 1.01-6.76, *p* = 0.045). Rs10069690 was associated with an increased risk of RCC under the dominant model. (OR=1.44, 95% CI= 1.04-2.01, *p* = 0.03). Haplotype “CA” was found to be associated with a decreased risk of RCC while haplotype “TA” was associated with an increased risk of RCC without adjustment for gender, age and body mass index (BMI). (OR=0.07; 95% CI= 0.01–0.54; *p*=0.011; OR= 1.24; 95% CI= 0.92–1.65; *p*=0.013, respectively). Rs35073794, rs10936599 and rs10069690 were positively correlated with the age older than 55 (OR= 3.27, 95%CI= 1.08-9.93, *p*=0.031; OR= 1.56, 95%CI= 1.03-2.37, *p=* 0.034; OR= 4.94, 95%CI= 1.18-20.70, *p=* 0.022, respectively) with or without history of drinking(OR= 4.47, 95%CI= 0.99-20.25, *p=* 0.024*;* OR= 2.62, 95%CI= 1.13-6.08, *p=* 0.022*;* OR=2.44, 95%CI=1.03-5.78, *p*= 0.04, respectively) and clinical stage I/II RCC (OR=2.62, 95%CI=1.02-6.74, *p*= 0.045; OR= 2.23, 95%CI= 1.08-4.60, *p=* 0.028; OR= 1.63, 95%CI= 1.17-2.27, *p*= 0.014, respectively). Our study indicated a significant association between SNPs in the *TERC*, *TERT* and RCC risk in a Chinese Han population. It could be used as diagnostic and prognostic markers in clinical studies of renal cell carcinoma patients.

## INTRODUCTION

Renal cell carcinoma (RCC) is a common malignant tumor of the urinary system, accounting for 3% of adult malignancies. In recent years, the incidence of renal cancer was increasing year by year. It is reported that there are about 209000 new cases of renal cell carcinoma and 102000 deaths per year in the world [1]. Many epidemiological studies indicated that environmental factors and life style including smoking, obesity, diesel exhaust, and various dioxins are involved in the development of renal cancer [2–4]. However, only some of patients who are exposed to these risk factors during their lifetime would finally develop renal cell carcinoma, which means that genetic susceptibility may play a role in the etiology of renal cancer. What's more, it is found that genetic mutations such as *VHL*, *PBRM1*, *FLCN* and *FH* are associated with renal cancer. [5–8].

Telomeres are located at the ends of chromosomes, they consist of tandem (TTAGGG)_n_ nucleotide repeats and some binding proteins [9]. The average telomere length is about 15 ~ 50 kB in human somatic cells, and shorten in most cells with aging [10]. Telomere plays a significant role in maintaining the stability and integrity of the genome [11]. Telomerase enzymes which including Telomerase Reverse Transcriptase (TERT), telomerase RNA component (TERC) are required to keep the maintenance of telomere [12]. Loss of telomere function and infinite proliferation leads to cell fusion, chromosome degradation and genetic instability, hence the cells could obtain further growth advantages, and ultimately develop into tumor cells [13].

Several association studies have observed that *TERT* and *TERC* had a role in susceptibility to tumorigenesis in multiple types of cancer, such as lung cancer, colon cancer, breast cancer, melanoma, thymic epithelial tumors and so on [14–20], which indicates mutations in *TERT* and *TERC* gene regions may affect the activities of telomerases and further affect the risk of cancers. Many studies have been performed to explore the associations between SNPs in the *TERT* and *TERC* genes and individual susceptibility to malignant tumors of the urinary system susceptibility, it is found that *TERT* rs2736100 AC genotype was associated with reduced risk of upper tract urothelial carcinomas [21], whereas AA genotype of rs2736098 was associated with an increased risk for RCC [22]. *TERC* rs10936599 was associated with an increased risk for bladder cancer [23]. Moreover, it is shown that *TERT* rs2242652 had a strong association with prostate cancer risk [24]. To further investigated correlations between the *TERT* and *TERC* polymorphisms and RCC susceptibility, we genotyped six SNPs in *TERT* and *TERC* genes: rs10936599 and rs35073794 on *TERC*; rs10069690, rs2242652, rs2853677 and rs285367 on *TERT*, and performed an association analysis to identify SNPs associated with RCC risk in Chinese Han population.

## RESULTS

### Participant characteristics

In our study, we recruited 293 patients with primary renal cell carcinoma and 459 healthy people. Basic characteristics of the control individuals and patients with renal cell cancer were shown in Table 1. There were statistical significance differences in age, gender and history of smoking and drinking between groups of case and control while no significant difference in BMI.

**Table 1:** Basic characteristics of the control individuals and patients with renal cell cancer

Characteristic	Case(N=293)	Controls(N=495)	*P*-value
Mean age ± SD	56.9±11.658 (N=292)	54.48±9.438 (N=495)	0.002^a^
Mean BMI ± SD	23.399±4.672(N=293)	23.087±3.156 (N=495)	0.311^a^
Gender			
male	193	180	0^b^
female	100	315	
Smoking			
yes	120	172	0^b^
no	173	173	
Drinking			
yes	53	59	0^b^
no	240	172	

SD: standard deviation; BMI: body mass index (weight [kg]/height[m] ^2^).

^a^*P* value was calculated by Welch's t test; ^b^*P* value was calculated by Pearson's χ 2 test.

0 cells (0.0%) have expected count less than 5.

### Hardy–Weinberg equilibrium test

Our study reveals that genotype distributions in cases and controls accorded with HWE for *TERC* gene rs35073794, rs10936599, *TERT* gene rs10069690, rs2242652, rs2853677, rs285367 sites, indicating that samples were representative.

### Association between genetic polymorphisms of *TERC* and *TERT* and RCC risk

Minor allele frequency (MAF) of each chosen SNP, detailed SNP data and the associations between various SNPs and RCC risk are shown in Table 2. Our research indicated that *TERC*-rs35073794 and *TERT-*rs10069690 were associated with an increased risk of RCC in an allele model (OR =2.39, 95% CI = 0.99-5.80, *p* = 0.047; OR =1.39, 95% CI = 1.07-1.81, *p* = 0.014, respectively).

**Table 2:** Candidate SNPs examined in TERC and TERT

SNP ID	Position	Band	Alleles A/B	Gene(s)	HWE-p	MAF		OR(95% CI)	*P*	*P*^’^
						Case	Control			
rs35073794	169482135	3q26.2	A/G	*TERC*	1	0.019	0.008	2.39(0.99-5.80)	0.047*	0.282
rs10936599	169492101	3q26.2	C/T	*TERC*	0.5834	0.456	0.435	1.09(0.89-1.32)	0.407	-
rs10069690	1279790	5p15.33	T/C	*TERT*	0.2659	0.189	0.143	1.39(1.07-1.81)	0.014*	0.084
rs2242652	1280028	5p15.33	A/G	*TERT*	1	0.188	0.164	1.19(0.92-1.53)	0.192	-
rs2853677	1287194	5p15.33	G/A	*TERT*	0.4397	0.382	0.368	1.06(0.87-1.30)	0.542	-
rs2853676	1288547	5p15.33	T/C	*TERT*	0.4932	0.179	0.156	1.19(0.91-1.54)	0.197	-

SNP: single nucleotide polymorphism; MAF: minor allele frequency; OR: odds ratio; 95% CI: 95% confidence interval.

* *p*≤ 0.05 indicates statistical significance; HWE: Hardy–Weinberg equilibrium.

*p* value were calculated using two-sided Chi-squared test.

p^’^value were calculated by the Bonferroni correction.

Five models including codominant, dominant, recessive, additive and genotype model were used to further assess the association between each SNP and RCC risk in a logistic regression analysis. The association between the SNPs and RCC risk in genotype model and codominat model was listed in Table 3. Due to the frequency of rs35073794 site's genotype “AA” was 0, we can't assess the association between rs35073794 and RCC risk in the recessive, and genotype model. We identified the genotype “TC” of rs10069690 was associated with an increased risk of RCC in the genotype model without adjustment for gender, age and BMI (OR =1.52, 95% CI = 1.11-2.08, *p* = 0.009). Rs3507794 was associated with an increased risk of RCC in the codominat model with adjustment for gender, age and BMI (OR =2.61, 95% CI = 1.01-6.76, *p* = 0.045). The minor allele of each SNP was regarded as a risk allele compared to the wild-type allele. Logistic tests were used to analyze further model association, as shown in Table 4, we found rs10069690 was associated with an increased risk of RCC under the dominant model with adjustment for gender, age and BMI (OR=1.44, 95% CI= 1.04-2.01, *p* = 0.03). In addition, no statistically significant difference was detected under the other models.

**Table 3:** The association between the single-nucleotide polymorphisms and RCC risk in genetype and codominant model

	Genetype	Case	Control	OR(95% CI)^1^	*P*1	OR(95% CI)^2^	*P*2
rs35073794	GG	280	487	NA	NA	1.00 [Ref]	0.045*
	AG	12	8	NA	NA	2.61(1.01-6.76)	
	AA	0	0	NA	NA	NA	
rs10936599	TT	80	161	1.00 [Ref]		1.00 [Ref]	0.24
	CT	158	237	1.32(0.96-1.81)	0.093	1.34 (0.94-1.90)	
	CC	54	97	1.13(0.75-1.70)	0.554	1.08 (0.69-1.70)	
rs10069690	CC	191	359	1.00 [Ref]		1.00 [Ref]	0.067
	TC	93	113	1.52(1.11-2.08)	0.009*	1.50 (1.06-2.11)	
	TT	8	13	1.37(0.60-3.11)	0.454	1.01 (0.39-2.57)	
rs2242652	GG	191	346	1.00 [Ref]		1.00 [Ref]	0.62
	AG	93	136	1.21(0.89-1.64)	0.222	1.18 (0.84-1.64)	
	AA	8	13	1.31(0.58-2.98)	0.516	0.95 (0.37-2.42)	
rs2853677	AA	112	202	1.00 [Ref]		1.00 [Ref]	0.54
	GA	132	222	1.03(0.76-1.39)	0.837	1.07 (0.77-1.49)	
	GG	48	71	1.15(0.76-1.74)	0.519	1.29 (0.82-2.04)	
rs2853676	CC	198	355	1.00 [Ref]		1.00 [Ref]	0.53
	TC	85	126	1.23(0.90-1.68)	0.191	1.23(0.90-1.68)	

SNP: single nucleotide polymorphism; OR: odd ratio; CI: confidence interval.

*p*1 value was calculated by logistic regression crude analysis.

*p*2 value was calculated by logistic regression analysis adjusted by Gender Age and BMI.

*p < 0.05 indicates statistical significance.

**Table 4:** Single loci association with renal cell carcinoma risk

SNP ID	Minor allele	Dominant model		Recessive model		Additive model	
		OR(95% CI)	*P*	OR(95% CI)	*P*	OR(95% CI)	*P*
rs35073794	A	2.61 (1.01-6.76)	0.045	NA	NA	NA	NA
rs10936599	C	1.26 (0.90-1.76)	0.17	0.90 (0.61-1.33)	0.6	1.07 (0.86-1.33)	0.54
rs10069690	T	1.44 (1.04-2.01)	0.03*	0.90 (0.35-2.28)	0.82	1.31 (0.98-1.74)	0.068
rs2242652	A	1.16 (0.84-1.60)	0.38	0.90 (0.35-2.29)	0.83	1.11 (0.83-1.47)	0.48
rs2853677	G	1.12 (0.82-1.53)	0.46	1.25 (0.82-1.90)	0.3	1.12 (0.90-1.40)	0.3
rs2853676	T	1.20 (0.86-1.66)	0.29	0.98 (0.40-2.38)	0.96	1.14 (0.86-1.52)	0.37

SNP: single nucleotide polymorphism; OR: odd ratio; CI: confidence interval.

*p* value was calculated by logistic regression analysis adjusted by Gender Age and BMI.

*p < 0.05 indicates statistical significance.

**Figure 1:**
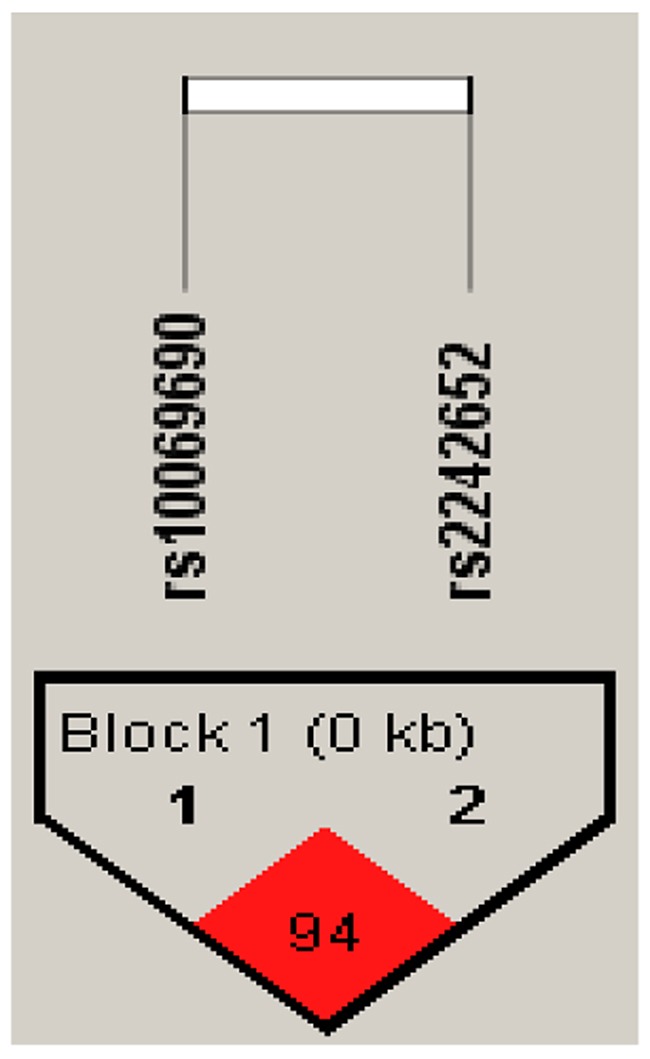
Haplotype block map for all the SNPs of the *TERT* gene

**Figure 2:**
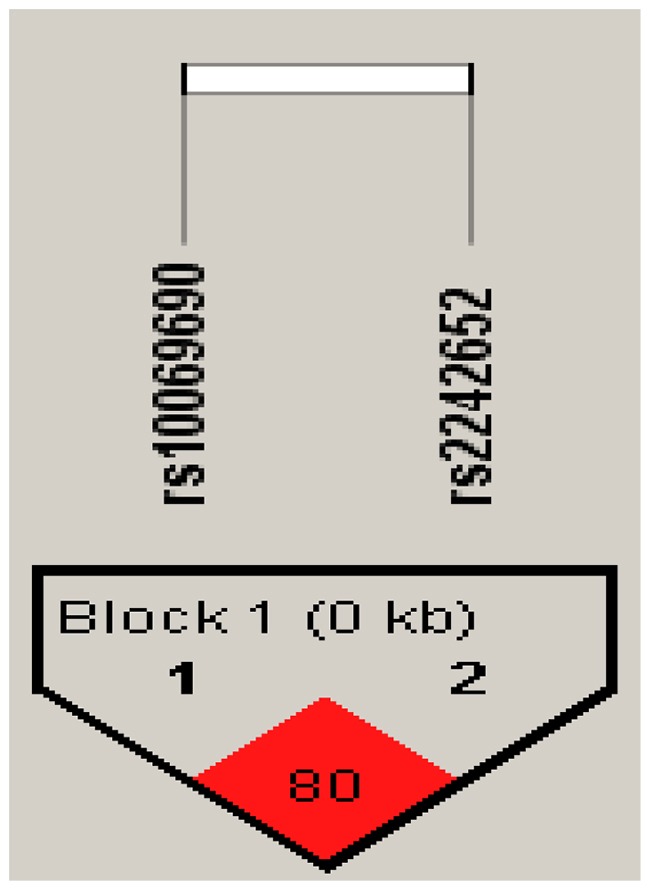
Haplotype block map for all the SNPs of the *TERT* gene

Furthermore, there is a strong linkage between the candidate SNPs in the *TERT* gene (Figures 1–2). According to assessing the associations between SNP haplotypes and RCC risk by performing unconditional logistic regression, we listed the results in Table 5. It is obvious that haplotype “TA” was associated with an increased risk of RCC without adjustment for gender, age and BMI (OR= 1.24; 95% CI= 0.92–1.65; *p*=0.013), however, the haplotype “CA” was found to be associated with a decreased risk of RCC with adjustment for gender, age and BMI (OR=0.07; 95% CI= 0.01–0.54; *p*=0.011).

**Table 5:** Haplotype frequency and their association with RCC risk in case and control subjects

SNPs	Haplotype	Freq%		*P*1	OR(95%CI)	*P*2
		Case	Control			
rs10069690|rs2242652	TA	0.194	0.144	0.013*	1.24 (0.92 - 1.65)	0.15
	CA	0	0.02	0.0018*	0.07 (0.01 - 0.54)	0.011*
	CG	0.804	0.83	0.2113	1	0.129

* *P*-value < 0.05 indicates statistical significance; OR: odd ratio; CI: confidence interval.

*P*1- values were calculated from two-sided Chi-squared test.

*P*2 -values were calculated by logistic regression analysis adjusted by gender & age & BMI.

*p < 0.05 indicates statistical significance.

Finally, the associations between *TERC* and *TERT* polymorphisms and clinical parameters of renal cell cancer had been further investigated. Positive results are shown in (Table 6A, 6B, 6C). By logistic regression crude analysis, it was found that the subjects whose age greater than 55, the genotype “AG” of rs35073794 was associated with an increased risk of RCC (OR= 3.27, 95%CI= 1.08-9.93, *p*=0.031). What's more, the genotype “AG” of rs35073794 was correlated with an increased risk of RCC when the variables were without history of drinking and clinical stages I/II (OR= 4.47, 95%CI= 0.99-20.25, *p=* 0.024*;* OR=2.62, 95%CI=1.02-6.74, *p*= 0.045, respectively). There were no significant differences with adjustment for gender, age and BMI. As for rs10936599 and rs10069690, by logistic regression crude analysis, it was found that the genotype “CT+CC” of rs10936599 and “CT+TT” of rs10069690 were associated with an increased risk of RCC when the subjects with a history of drinking (OR= 2.62, 95%CI= 1.13-6.08, *p=* 0.022*;* OR=2.44, 95%CI=1.03-5.78, *p*= 0.04, respectively). Subjects who older than 55 years of age carried the genotype “CT+CC” of rs10936599 was a risk factor of RCC without adjustment for gender, age and BMI (OR= 1.56, 95%CI= 1.03-2.37, *p=* 0.034), the genotype “CT+TT” of rs10069690 was also a risk factor of RCC with adjustment for gender, age and BMI (OR= 4.94, 95%CI= 1.18-20.70, *p=* 0.022). When the variable was clinical stages I/II, the genotype “CT+CC” of rs10936599 was associated with an increased risk of RCC with adjustment for gender, age and BMI (OR= 2.23, 95%CI= 1.08-4.60, *p=* 0.028), the genotype “CT+TT” of rs10069690 were likely a risk factor of RCC by logistic regression crude analysis (OR= 1.63, 95%CI= 1.17-2.27, *p*= 0.014).

**Table 6A:** The associations between TERC and TERT polymorphisms and clinical characteristics of renal cell cancer

Variables	rs35073794						
	Genotype	Case	Control	OR(95%CI)^a^	*P*^a^	OR(95%CI)^b^	*P*^b^
Age							
<55	GG	120	196	1	0.55	1	0.27
	AG	3	3	1.63 (0.32-8.22)		3.04 (0.45-20.46)	
≥55	GG	160	291	1	0.031*	1	0.067
	AG	9	5	3.27 (1.08-9.93)		0.03 (0.00-1.64)	
Smkoing							
yes	GG	113	57	1	0.47	1	1
	AG	7	2	1.77 (0.36-8.77)		7.88 (0.00-NA)	
no	GG	168	170	1	0.25	1	0.65
	AG	5	2	2.53 (0.48-13.22)		2.15 (0.08-59.64)	
Drinking							
yes	GG	53	57	1	0.11	1	1
	AG	2	0	0.00(0.00-NA)		1.34 (0.00-NA)	
no	GG	228	170	1	0.024*	1	0.89
	AG	12	2	4.47 (0.99-20.25)		1.28 (0.04-44.58)	
Clinical Stages							
I/II	GG	232	487	1	0.045*	1	0.67
	AG	10	8	2.62 (1.02-6.74)		1.61 (0.17-15.06)	
III/IV	GG	47	487	1	0.28	1	0.4
	AG	2	8	2.59 (0.53-12.55)		4.79 (0.12-185.23)	

**Table 6B:** The associations between TERC and TERT polymorphisms and clinical characteristics of renal cell cancer

Variables	rs10936599						
	Genotype	Case	Control	OR(95%CI)^a^	*P*^a^	OR(95%CI)^b^	*P*^b^
Age							
<55	TT	36	56	1	0.83	1	0.81
	CT+CC	87	143	0.95 (0.58-1.55)		0.92 (0.47-1.80)	
≥55	TT	44	105	1	0.034*	1	0.28
	CT+CC	125	191	1.56 (1.03-2.37)		2.03 (0.55-7.47)	
Smkoing							
yes	TT	30	23	1	0.057	1	0.096
	CT+CC	90	36	1.92 (0.98-3.73)		0.00 (0.00-NA)	
no	TT	51	57	1	0.46	1	0.94
	CT+CC	122	115	1.19 (0.75-1.87)		1.04 (0.37-2.98)	
Drinking							
yes	TT	11	24	1	0.022*	1	0.13
	CT+CC	42	35	2.62 (1.13-6.08)		6.25 (0.53-73.44)	
no	TT	70	56	1	0.46	1	0.38
	CT+CC	170	116	1.17 (0.77-1.79)		1.47 (0.62-3.50)	
Clinical Stages							
I/II	TT	65	161	1	0.11	1	0.028*
	CT+CC	177	334	1.31 (0.93-1.85)		2.23 (1.08-4.60)	
III/IV	TT	15	161	1	0.78	1	0.98
	CT+CC	34	334	1.09 (0.58-2.06)		0.98 (0.23-4.11)	

**Table 6C:** The associations between TERC and TERT polymorphisms and clinical characteristics of renal cell cancer

Variables	rs10069690						
	Genotype	Case	Control	OR(95%CI)^a^	*P*^a^	OR(95%CI)^b^	*P*^b^
Age							
<55	CC	85	142	1	0.48	1	0.82
	CT+TT	38	53	1.20 (0.73-1.97)		1.08 (0.55-2.12)	
≥55	CC	106	217	1	0.0065*	1	0.022*
	CT+TT	63	73	1.77 (1.17-2.66)		4.94 (1.18-20.70)	
Smkoing							
yes	CC	76	43	1	0.15	1	0.096
	CT+TT	44	15	1.66 (0.83-3.33)		0.00 (0.00-NA)	
no	CC	115	125	1	0.13	1	0.53
	CT+TT	58	44	1.43 (0.90-2.28)		1.42 (0.48-4.21)	
Drinking							
yes	CC	34	48	1	0.04*	1	0.25
	CT+TT	19	11	2.44 (1.03-5.78)		5.88 (0.25-140.94)	
no	CC	157	120	1	0.2	1	0.99
	CT+TT	83	48	1.32 (0.86-2.03)		1.01 (0.40-2.57)	
Clinical Stages							
I/II	CC	154	359	1	0.014*	1	0.25
	CT+TT	88	126	1.63 (1.17-2.27)		1.51 (0.75-3.06)	
III/IV	CC	36	359	1	0.93	1	0.96
	CT+TT	13	126	1.03 (0.53-2.00)		0.96 (0.20-4.63)	

CI: confidence interval; OR: odds ratio; ^a^logistic regression crude analysis.

^b^logistic regression analysis adjusted by gender, age and BMI; *p < 0.05 indicates statistical significance.

## DISCUSSION

We investigated the associations between SNPs in *TERC*, *TERT* gene and risk of RCC in Chinese Han population in this case–control study. It is indicated that three SNPs are related to RCC: *TERC*-rs35073794 and *TERT* -rs10069690 were associated with an increased risk of RCC The further analysis of associations showed that the *TERT* gene haplotype “TA” was associated with an increased risk of RCC, while the haplotype “CA” was found to be associated with a decreased risk of RCC. Finally, rs35073794, rs10936599 and rs10069690 were positively correlated with the age older than 55, with or without history of drinking and clinical stage I/II RCC.

We are the first to demonstrate an association between rs35073794 and RCC susceptibility. Meanwhile, we revealed an association between rs1069690 and RCC susceptibility in Chinese Han population. Interestingly, Michela de Martino et al. showed that there was no association between rs1069690 and risk of RCC in European [22]. As it is reported that distribution of SNPs, the haplotype and linkage disequilibrium features in races and ethnic groups are different [25, 26], the different conclusions about the association between rs10069690 and RCC may be explained by distribution of the SNPs among races and ethnic groups.

Telomere achieves its’ genomic integrity and stability maintained by preventing the chromosome from shortening and losing genetic data with chromosomal replication. Meanwhile, in the role of DNA polymerase, telomere gradually shrinks during mitosis. When telomeres are too short to carry out their function, cells tend to senescence and apoptosis [27]. However, in tumor cells, telomeres avoid shortening by abnormal activation of telomerases, hence tumor cells would keep proliferating [28]. It is reported that SNPs in telomerase-associated *TERT* and *TERC* gene are associated with telomere length. Andrew J Pellatt et al. indicated *TERT* rs2853676 was associated with telomere length. Use of aspirin/NSAIDs interacted significantly with *TERT* rs10069690 to alter telomere length [15]. Codd V et al. and Soerensen M have reported an association between telomere length and genetic variations in *TERC,* it was found rs10936599 in TERC involved in telomere biology by affecting mean telomere length [29–31], while Mirabello et al. did not find an association between *TERC* and telomere length [11]. Interestingly, Kathryn L et al. observed several SNPs in the *TERT* gene, rs2736122, rs4246742, rs4975605, rs10069690, rs2736100, rs2853676, rs7726159 were significantly associated with ovarian cancer risk. However, there were no differences in telomere length between cases and controls [32], and Hosen I et al. measured relative telomere length in clear cell renal cell carcinoma and difference between tumors with and without the *TERT* promoter mutations was not statistically significant [33]. The pathogenesis between telomerase-associated gene mutations and RCC is still unclear and needed to be further investigated, and there might be other telomerase-associated pathogenesis could cause cancers.

Rs10069690 which is mapped to intron 4 of *TERT* gene was located on 5p15.33. It was associated with many caners such as glioblastoma, breast cancer, ovarian and so on [18, 33, 34]. In some malignant tumors of the urinary system, rs10069690 was found associated with prostate cancer, in our study, rs10069690 was associated with an increased risk of RCC, but it was found to be significantly associated with a decreased risk for an aggressive form of prostate cancer in Chinese population [35]. Dong J and colleagues demonstrated that the non-T allele of rs10069690 may increase the risk of primary hepatocellular carcinoma in Chinese population [36]. Such notable contradictions might be explained by the complexity of interactions among genetic variations, telomere stabilities and structures, clinical phenotypes and so on. Furthermore, to the best of our knowledge, there are few articles on the association between rs35073794 and risk of tumors. Rs35073794 lies in downstream of *TERC* and it is located on 3q26.2. In our study, rs35073794 was increased 2.39-fold RCC susceptibility by the allele model. This site in *TERC* needed to be further investigated to research correlations between rs35073794 and cancers. It was showed that rs10936599 located on 3q26.2 was correlated with a decreased risk of colorectal cancer [37]. However, in our study, rs10936599 was found to be a risk factor of RCC. As mentioned above, rs10936599 may be involved in the pathogeneses of malignant neoplasm by altering the length of telomere while the specific pathogenesis needs to be further explored.

This study has some potential limitations. First of all, this study is limited by its sample size, the further correlation should be confirmed by performing a large sample size meta-analysis; Secondly, clinical characteristics including tumor size and extent of aggression were not included in our study, and it is needed to be further analyzed through additional studies. What's more, additional studies should be performed with more environmental, and life style factors considered. Last, our experiments were not designed to investigate the relationship between telomere length and RCC, and the detail pathogenesis between telomerase-associated gene and RCC is still not clear, the function genetic variants and mechanisms should be further investigated.

To sum up, we have demonstrated that three SNPs (rs35073794, rs10936599, rs10069690) in *TERC* and *TERT* gene are associated with risk of RCC in Chinese Han population for the first time. Of course, the influence by lifestyle can't be underestimated. Our study may provide new data for screening of RCC in Han population and could be used as diagnostic and prognostic markers in clinical studies of renal cell carcinoma patients.

## MATERIALS AND METHODS

### Research objects

A total of 293 patients with primary renal cell carcinoma and 459 healthy people were recruited from 2011 to 2016 among Shaanxi Province. All the patients were treated by the First Affiliated Hospital of Xi’an Jiaotong University and were newly diagnosed renal cell carcinoma by the pathological examination. Among the 293 patients, there were two specific subtypes including renal clear cell carcinoma (291/293) and renal papillary cell carcinoma (2/293). Patients who had not yet received any chemotherapy or radiotherapy were included for the case group. People who suffered from kidney insufficiency or had hereditary cancer syndrome history were excluded. 459 healthy unrelated subjects were recruited randomly as control group, individuals are Han Chinese living Xi’an. Moreover, people with chronic disease involving brain, liver, heart, and lung were excluded from our study. Individuals with urinary system diseases were also excluded. All samples were collected with informed consent and the study was approved by the regional ethics committee.

### SNP selection and genotyping

We reviewed the literatures related to association between *TERT* and *TERC* polymorphisms and tumors of urinary system and selected SNPs in *TERT* and *TERC* with the minor allele frequencies (MAF) ≥5% in Asian by using HapMap database [22–24, 38]. In addition, the correlation between chosen SNPs and RCC in Chinese Han population has not been reported before. Genomic DNA was extracted from whole blood samples using the Gold Mag-Mini Whole Blood Genomic DNA Purification Kit (version 3.0; TaKaRa, Japan) [39]. The DNA concentration was measured by spectrometry (DU530 UV/VIS spectrophotometer, Beckman Instruments, Fullerton, CA, USA). The Sequenom MassARRAY Assay Design 3.0 software (Sequenom, Inc, San Diego, CA, USA) was used to design the multiplexed SNP Mass EXTEND assay. Genotyping was performed using a Sequenom MassARRAY RS1000 (Sequenom, Inc.) in accordance with the manufacturer's protocol. Sequenom Typer 4.0 software was used to perform data management and analyses [40]. The primers corresponding to each SNP are listed in Table 7. Based on these results, six SNPs including rs35073794, rs10936599, rs10069690, rs2242652, rs2853677, rs2853676 were selected.

**Table 7:** Primers used for this study

SNP_ID	1st-PCRP	2nd-PCRP	UEP_SEQ
rs35073794	ACGTTGGATGGTCTTCCGCTTTTTGTTGCC	ACGTTGGATGAGAAGCAAAAACCTCAACA	cctCAAAAACCTCAACAAAATCT
rs10936599	ACGTTGGATGTTCCCGCTGTTTGTTCAGTC	ACGTTGGATGCAAGGGTAAAATTCCATTCTG	ATGCAGTATTCGCACCA
rs10069690	ACGTTGGATGCCTGTGGCTGCGGTGGCTG	ACGTTGGATGATGTGTGTTGCACACGGGAT	GGGATCCTCATGCCA
rs2242652	ACGTTGGATGACAGCAGGACACGGATCCAG	ACGTTGGATGAGGCTCTGAGGACCACAAGA	gtcgGAGGACCACAAGAAGCAGC
rs2853677	ACGTTGGATGATCCAGTCTGACAGTCGTTG	ACGTTGGATGGCAAGTGGAGAATCAGAGTG	gggtAATCAGAGTGCACCAG
rs2853676	ACGTTGGATGTGTCTCCTGCTCTGAGACC	ACGTTGGATGCAAAACTAAGACCCAAGAGG	agatGGAAGTCTGACGAAGGC

### Statistical analysis

We performed a two-sided Chi-squared test to examine Hardy-Weinberg equilibrium (HWE) in case and control groups. All of the minor alleles were deemed as a risk allele for RCC susceptibility. The differences in frequency distributions of alleles were compared between cases and controls by two-sided Chi-squared test. Odds ratios (ORs), 95% confidence intervals (CIs) and *p*-value were used for crude logistic regression analysis and logistic regression analysis adjusted by gender, age and BMI. We used the Haploview software package (version 4.2) and the SHEsi software platform to analyze the linkage disequilibrium and SNP haplotypes [41, 42]. SPSS version 22.0 statistical package (SPSS, Chicago, IL, USA) and Microsoft Excel were used for all statistical analyses. P<0.05 was considered statistically significant.

## Abbreviations

SNP: single nucleotide polymorphism
RCC: renal cell carcinoma
TERT: telomerase reverse transcriptase
TERC: telomerase RNA component
BMI: body mass index (weight [kg]/height[m] ^2^)
MAF: minor allele frequency
OR: odds ratio
95% CI: 95% confidence interval

## References

[1] Rini BI, Campbell SC, Escudier B (2009). Renal cell carcinoma. Lancet.

[2] Theis RP, Dolwick Grieb SM, Burr D, Siddiqui T, Asal NR (2009). Smoking, environmental tobacco smoke, and risk of renal cell cancer: a population-based case-control study. BMC Cancer.

[3] Kroeger N, Klatte T, Birkhäuser FD, Rampersaud EN, Seligson DB, Zomorodian N, Kabbinavar FF, Belldegrun AS, Pantuck AJ (2012). Smoking negatively impacts renal cell carcinoma overall and cancer-specific survival. Cancer.

[4] Mclaughlin JK, Gao YT, Gao RN, Zheng W, Ji BT, Blot WJ, Fraumeni JF (1992). Risk factors for renal-cell cancer in Shanghai, China. Int J Cancer.

[5] Dalgliesh GL, Furge K, Greenman C, Chen L, Bignell G, Butler A, Davies H, Edkins S, Hardy C, Latimer C, Teague J, Andrews J, Barthorpe S (2010). Systematic sequencing of renal carcinoma reveals inactivation of histone modifying genes. Nature.

[6] Varela I, Tarpey P, Raine K, Huang D, Ong CK, Stephens P, Davies H, Jones D, Lin ML, Teague J, Bignell G, Butler A, Cho J (2011). Exome sequencing identifies frequent mutation of the SWI/SNF complex gene PBRM1 in renal carcinoma. Nature.

[7] Toro JR, Wei MH, Glenn GM, Weinreich M, Toure O, Vocke C, Turner M, Choyke P, Merino MJ, Pinto PA, Steinberg SM, Schmidt LS, Linehan WM (2008). BHD mutations, clinical and molecular genetic investigations of Birt–Hogg–Dubé syndrome: a new series of 50 families and a review of published reports. J Med Genet.

[8] Tomlinson IP, Alam NA, Rowan AJ, Barclay E, Jaeger EE, Kelsell D, Leigh I, Gorman P, Lamlum H, Rahman S, Roylance RR, Olpin S, Bevan S (2002). Germline mutations in FH predispose to dominantly inherited uterine fibroids, skin leiomyomata and papillary renal cell cancer. Nat Genet.

[9] Moon IK, Jarstfer MB (2007). The human telomere and its relationship to human disease, therapy, and tissue engineering. Front Biosci.

[10] Aubert G, Lansdorp PM (2008). Telomeres and aging. Physiol Rev.

[11] Mirabello L, Yu K, Kraft P, De Vivo I, Hunter DJ, Prescott J, Wong JY, Chatterjee N, Hayes RB, Savage SA (2010). The association of telomere length and genetic variation in telomere biology genes. Hum Mutat.

[12] Greider CW (1998). Telomerase activity, cell proliferation, and cancer. Proc Natl Acad Sci U S A.

[13] Lin TT, Letsolo BT, Jones RE, Rowson J, Pratt G, Hewamana S, Fegan C, Pepper C, Baird DM (2010). Telomere dysfunction and fusion during the progression of chronic lymphocytic leukemia: evidence for a telomere crisis. Blood.

[14] Liu SG, Ma L, Cen QH, Huang JS, Zhang JX, Zhang JJ (2015). Association of genetic polymorphisms in TERT-CLPTM1L with lung cancer in a Chinese population. Genet Mol Res.

[15] Pellatt AJ, Wolff RK, Lundgreen A, Cawthon R, Slattery ML (2011). Genetic and lifestyle influence on telomere length and subsequent risk of colon cancer in a case control study. Int J Mol Epidemiol Genet.

[16] Huang DS, Wang Z, He XJ, Diplas BH, Yang R, Killela PJ, Meng Q, Ye ZY, Wang W, Jiang XT, Xu L, He XL, Zhao ZS (2015). Recurrent TERT promoter mutations identified in a large-scale study of multiple tumour types are associated with increased TERT expression and telomerase activation. Eur J Cancer.

[17] Gibbs DC, Orlow I, Kanetsky PA, Luo L, Kricker A, Armstrong BK, Anton-Culver H, Gruber SB, Marrett LD, Gallagher RP, Zanetti R, Rosso S, Dwyer T (2015). Inherited genetic variants associated with occurrence of multiple primary melanoma. Cancer Epidemiol Biomarkers Prev.

[18] Haiman CA, Chen GK, Vachon CM, Canzian F, Dunning A, Millikan RC, Wang X, Ademuyiwa F, Ahmed S, Ambrosone CB, Baglietto L, Balleine R, Bandera EV (2011). A common variant at the TERT-CLPTM1L locus is associated with estrogen receptor-negative breast cancer. Nat Genet.

[19] Li X, Xu X, Fang J, Wang L, Mu Y, Zhang P, Yao Z, Ma Z, Liu Z (2016). Rs2853677 modulates Snail1 binding to the TERT enhancer and affects lung adenocarcinoma susceptibility. Oncotarget.

[20] Jin TB, Zhang JY, Li G, Du SL, Geng TT, Gao J, Liu QP, Gao GD, Kang LL, Chen C, Li SQ (2013). RTEL1 and TERT polymorphisms are associated with astrocytoma risk in the Chinese Han population. Tumour Biol.

[21] Yuan X, Meng Y, Li P, Ge N, Kong F, Yang L, Björkholm M, Zhao S, Xu D (2016). The association between the TERT rs2736100 AC genotype and reduced risk of upper tract urothelial carcinomas in a Han Chinese population. Oncotarget.

[22] de Martino M, Taus C, Lucca I, Hofbauer SL, Haitel A, Shariat SF, Klatte T (2015). Association of human telomerase reverse transcriptase gene polymorphisms, serum levels, and telomere length with renal cell carcinoma risk and pathology. Mol Carcinog.

[23] Wang M, Chu H, Lv Q, Wang L, Yuan L, Fu G, Tong N, Qin C, Yin C, Zhang Z, Xu J (2014). Cumulative effect of genome-wide association study-identified genetic variants for bladder cancer. Int J Cancer.

[24] Kote-Jarai Z, Saunders EJ, Leongamornlert DA, Tymrakiewicz M, Dadaev T, Jugurnauth-Little S, Ross-Adams H, Al Olama AA, Benlloch S, Halim S, Russell R, Dunning AM, Luccarini C (2013). Fine-mapping identifies multiple prostate cancer risk loci at 5p15, one of which associates with TERT expression. Hum Mol Genet.

[25] Taillon-Miller P, Gu Z, Li Q, Hillier L, Kwok PY (1998). Overlapping genomic sequences: a treasure trove of single-nucleotide polymorphisms. Genome Res.

[26] Tang K, Wong LP, Lee EJ, Chong SS, Lee CG (2004). Genomic evidence for recent positive selection at the human MDR1 gene locus. Hum Mol Genet.

[27] Verfaillie CM, Pera MF, PM Lansdorp (2002). Stem cells: hype and reality. Hematology.

[28] Hiyama E, Hiyama K (2003). Telomerase as tumor marker. Cancer Lett.

[29] Codd V, Mangino M, van der Harst P, Braund PS, Kaiser M, Beveridge AJ, Rafelt S, Moore J, Nelson C, Soranzo N, Zhai G, Valdes AM, Blackburn H (2010). Common variants near TERC are associated with mean telomere length. Nat Genet.

[30] Soerensen M (2012). Genetic variation and human longevity. Dan Med J.

[31] Codd V, Nelson CP, Albrecht E, Mangino M, Deelen J, Buxton JL, Hottenga JJ, Fischer K, Esko T, Surakka I, Broer L, Nyholt DR, Mateo Leach I (2013). Identification of seven loci affecting mean telomere length and their association with disease. Nat Genet.

[32] Terry KL, Tworoger SS, Vitonis AF, Wong J, Titus-Ernstoff L, De Vivo I, Cramer DW (2012). Telomere length and genetic variation in telomere maintenance genes in relation to ovarian cancer risk. Cancer Epidemiol Biomarkers Prev.

[33] IHosen I, Rachakonda PS, Heidenreich B, Sitaram RT, Ljungberg B, Roos G, Hemminki K, Kumar R (2015). TERT promoter mutations in clear cell renal cell carcinoma. Int J Cancer.

[34] Mosrati MA, Malmström A, Lysiak M, Krysztofiak A, Hallbeck M, Milos P, Hallbeck AL, Bratthäll C, Strandéus M, Stenmark-Askmalm M, Söderkvist P (2015). TERT promoter mutations and polymorphisms as prognostic factors in primary glioblastoma. Oncotarget.

[35] Wu D, Yu H, Sun J, Qi J, Liu Q, Li R, Zheng SL, Xu J, Kang J (2015). Association of genetic polymorphisms in the telomerase reverse transcriptase gene with prostate cancer aggressiveness. Mol Med Rep.

[36] Dong J, Wang L, Tian YP, Guo Y, Liu HY (2011). [hTERT single nucleotide polymorphism is associated with increased risks of hepatocellular carcinoma and tumor metastasis]. [Article in Chinese]. Nan Fang Yi Ke Da Xue Xue Bao.

[37] Houlston RS, Cheadle J, Dobbins SE, Tenesa A, Jones AM, Howarth K, Spain SL, Broderick P, Domingo E, Farrington S, Prendergast JG, Pittman AM, Theodoratou E (2010). Meta-analysis of three genome-wide association studies identifies susceptibility loci for colorectal cancer at 1q41, 3q26.2, 12q13.13 and 20q13.33. Nat Genet.

[38] Li T, Xian Y, Tian T, Zhuang X, Chu M (2016). New evidence of TERT rs2736098 polymorphism and cancer risk: an updated meta-analysis.

[39] Carracedo A (2007). Forensic DNA Typing Protocols.

[40] Gabriel S, Ziaugra L, Tabbaa D (2009). SNP genotyping using the. Sequenom MassARRAY iPLEX platform.

[41] Barrett JC, Fry B, Maller J, Daly MJ (2005). Haploview: analysis and visualization of LD and haplotype maps. Bioinformatics.

[42] Shi YY, He L (2005). SHEsis, a powerful software platform for analyses of linkage disequilibrium, haplotype construction, and genetic association at polymorphism loci. Cell Res.

